# Apalutamide for non-metastatic castration-resistant prostate cancer (nmCRPC): real world data of a multicenter study

**DOI:** 10.1007/s00432-024-05928-7

**Published:** 2024-09-09

**Authors:** Axel Hegele, Rainer Häußermann, Stefan Schultheis, Lennart Skrobek, Meike Vink, Sebastian Hollwegs, Martin Ludwig, Petra Huwe, Manfred Maywurm, Anke Bartsch-Polle, Jost Weber, Markus Thiemer, Denny Varughese

**Affiliations:** 1 Urological Center Mittelhessen, DRK Hospital Biedenkopf, Biedenkopf, Germany; 2 Department of Urology, Philipps University Marburg, Marburg, Germany; 3 Urologen am Ludwigsplatz, Giessen, Germany; 4 Urological Center Wetterau, Büdingen, Germany; 5 Urological Practice, Homberg/Efze, Germany; 6 UroFaz, Wetzlar, Germany; 7 Urological Center Marburg, Marburg, Germany; 8 Urological Practice, Giessen, Germany; 9 Urological practice clinic Marburg, Marburg, Germany; 10 Department of Radiotherapy and Radiooncology, Philipps-University Marburg, Marburg, Germany

**Keywords:** Real-world data, Non-metastatic castration-resistant prostate cancer, Apalutamide, Metastasis-free survival

## Abstract

**Purpose:**

Apalutamide plus androgen-deprivation therapy (ADT) improved outcomes in patients with non-metastatic castration-resistant prostate cancer (nmCRPC). Nevertheless real-world data are limited. The aim of this multicenter study was to generate real-world data from nmCRPC patients treated with ADT plus apalutamide.

**Methods:**

In this observational cohort based investigator initiated trial data of nmCRPC patients receiving apalutamide plus ADT were collected focusing on patient demographic data, prostate-specific antigen (PSA) declines, safety profile including dose modification/discontinuation as well as subsequent therapy and metastasis-free survival (MFS).

**Results:**

Data from a total of 31 nmCRPC patients were documented. Compared to the Phase III study Spartan real-world patients are older, showed a higher ECOG-PS and more aggressive tumors. ***In the cohort*** PSA decreased about 98.1%, ***74%*** of patients showed a PSA decrease ***over 90%*** and 54.8% reached a PSA-level < 0.2ng/ml. Apalutamide was well tolerated in real world patients: adverse events occurred in 67.7% but were in the majority mild (≥ grade 3: 6.5%). Dose reduction was necessary in 38.7% and 32.2% discontinued apalutamide treatment. MFS was 43 months and majority of patients were subsequently treated with abiraterone.

**Conclusion:**

In real world more comorbid nmCRPC patients with a higher ECOG-PS and more aggressive tumors are treated with apalutamide plus ADT. Nevertheless efficacy results as well as side effects are similar in real-world compared to Spartan trial showing also a rapid, durable and deep PSA response with a median MFS of 43 months.

## Introduction

Patients with non-metastatic castration-resistant prostate cancer (nmCRPC) without further treatment are developing metastases associated with significant morbidity and mortality ***despite*** ongoing androgen-deprivation therapy (ADT) (Scher 2015, Crona 2017; Smith 2011). A short PSA doubling time (PSADT) ≤ 10 months and a PSA-level ≥ 8ng/ml may help to identify nmCRPC patients who were at high risk for disease progression (Smith 2011 and 2013). In 2018 the Phase III trial Spartan showed that the oral non-steroidal anti-androgen apalutamide increased metastasis-free survival (MFS) as well as overall survival (OS) in high-risk nmCRPC patients compared to placebo leading to approval of apalutamide for patients with high-risk nmCRPC (Smith 2018 and 2021). Apalutamide binds directly to the ligand-binding domain of the androgen-receptor (AR) thus preventing AR translocation, DNA binding and transcription mediated by AR (Clegg 2012). Results of randomized clinical trials (RCT) represents the highest level of evidence (Dahm 2020). Nevertheless RCTs have limitations – most notably used inclusion criteria often do not reflect real-world patient population. Elderly and more comorbid patients were common especially in cases of prostate cancer. To date patients of this type are often underrepresented or even not represented at all in clinical trials. So real-world data adding valuable data to the information obtained from RCTs are of high interest (Schad 2022; Baumfeld 2020). The aim of our study was to generate real-world data in nmCRPC patients treated with apalutamide in daily practice concerning patient population, used imaging tools, efficacy, safety as well as dosage-changes and subsequent therapy.

## Materials and methods

This study (Use of **A**paluta**m**ide in **p**rostate cancer in C**e**ntra**l** Hessen: AmPel) is an observational ***retrospective*** cohort based investigator initiated trial (IIT) with focus on real world evidence. The study was performed from November 2021 (ongoing) in nine urological practices in the middle of Hessen/Germany. Patients who started apalutamide therapy due to nmCRPC were added and followed as commonly done in each practice ***without a universal protocol and specifications concerning imaging controls and time intervals.***

The study followed the principles of the Declaration of Helsinki and was approved by the local ethics committee of the Philipps University Marburg, Medicine school (authorization number: 163/21).

Patients data included age, time of follow-up, ECOG performance Score (ECOG PS), date of initial prostate-cancer diagnosis, Gleason-Score, initial PSA-level, prior cancer-specific treatment, time to castration resistance, PSADT as well as used imaging method.

Efficacy variables included PSA levels prior start of apalutamide treatment (baseline) and during treatment – normally all 3 months like it was performed routinely in the participating urological practices. Decrease of PSA levels after time and best achieved ***individual*** percentage PSA-decrease ***(≥ 50% PSA reduction***,*** ≥ 90% PSA reduction)*** as well as patient reaching a PSA ≤ 0.2ng/ml were estimated.

Discontinuation of treatment due to various reasons (progression, toxicity, patients request) and subsequent therapies were documented. MFS was calculated.

Safety variables were the type of adverse event (AE) and the respective grade according to the National Cancer Institute Common Terminology Criteria for Adverse Events (NCI-CTCAE, version 5.0).

Data were collected and analyzed using Excel for Windows (Version 2013). Kaplan Meier survival analysis on MFS was performed with Graphpad Prism 9.5.1.

## Results

### Patients

In this ongoing IIT data was analyzed in a total of 31 nmCRPC patients and documented from November 2021 until November 2023. These patients were included in 9 urological practices in Hessen/Germany. ***39.1% of the patients underwent previous prostate cancer treatment (prostatectomy***,*** radiation). The median PSA-level before starting apalutamide therapy was 7.21 ng/ml***,*** median PSA doubling time (PSADT) was 4 months and 81.5% showed a PSADT ≤ 6 month.*** All patients started with the full apalutamide dosage of 240 mg daily. Median follow-up was 18 months (range 3–50). Median time to castration resistance was 72 months (range 12–264). Patients and disease characteristics before starting apalutamide treatment are summarized in Table 1 in comparison to the pivotal trial Spartan (Smith 2018).

**Table 1:** Characteristics of nmCRPC patients compared to spartan trial

	AmPel trial	Spartan trial
**Number of patients [n]**	31	806
**Median FU [months]**	18	20.3
**Median age [years, range]**	78 (56–89)	74 (52–97)
**ECOG PS [%]**		
*0*	29.6	77.3
*1*	40.7	22.7
*2*	25.9	0
*3*	3.7	0
**Previous cancer treatment [%]**		
*Prostatectomy/Radiation*	39.1	76.6
**Gleason Score [%]**		
*< 7*	10.5	19.4
*= 7*	31.6	37.1
*> 7*	57.9	43.5
**Diagnostic technique [%]**		
*Conventional imaging*	96.8	100
*PSMA-PET/CT*	3.2	0
**PSA doubling time (months)**		
*Median*	4.0	4.4
*≤ 6 months [%]*	81.5	71.5
*> 6 months [%]*	18.5	28.5
**median PSA baseline [ng/ml]**	7.21	7.78

### Efficacy

**Fig. 1:**
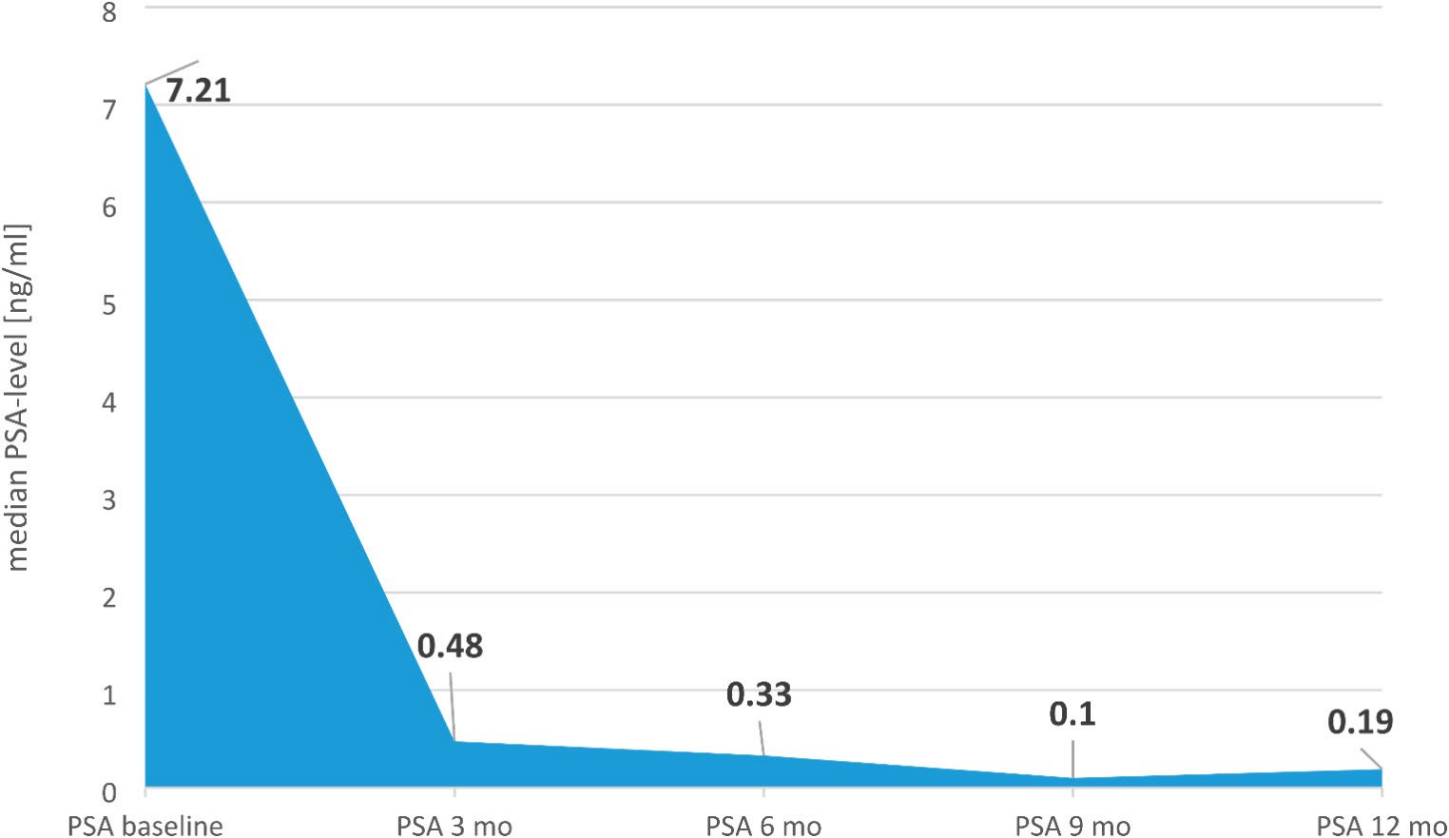
Median PSA levels of the cohort at different time points after starting apalutamide treatment showing a rapid and durable PSA response

**Fig. 2:**
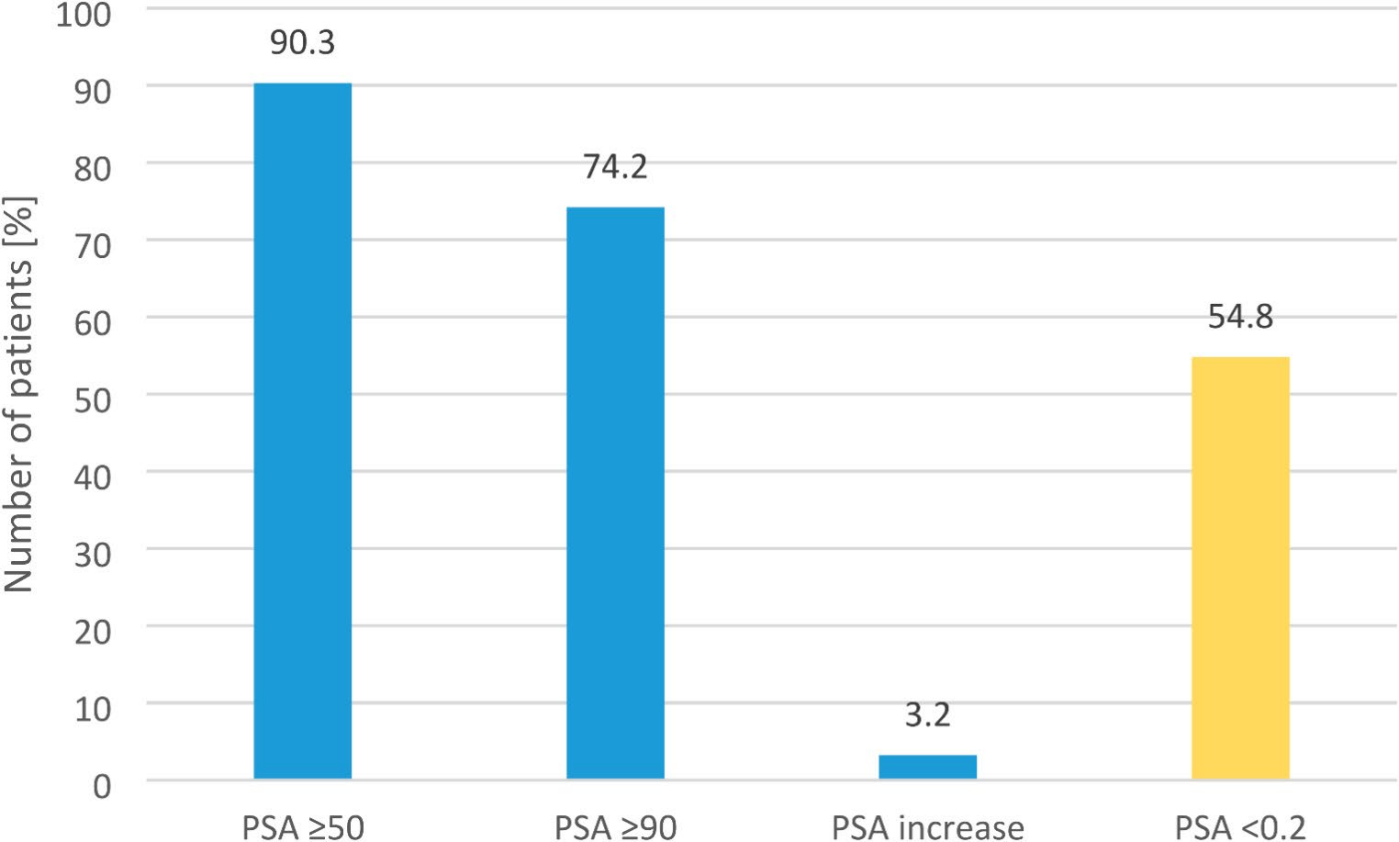
Number of patients [%] who achieved decreased PSA-levels ≥ 50%, ≥ 90% and PSA increase (blue bars) after 3 months. Yellow bar show the number of patients [%] with PSA decrease ≤ 0.2 ng/ml

**Fig. 3:**
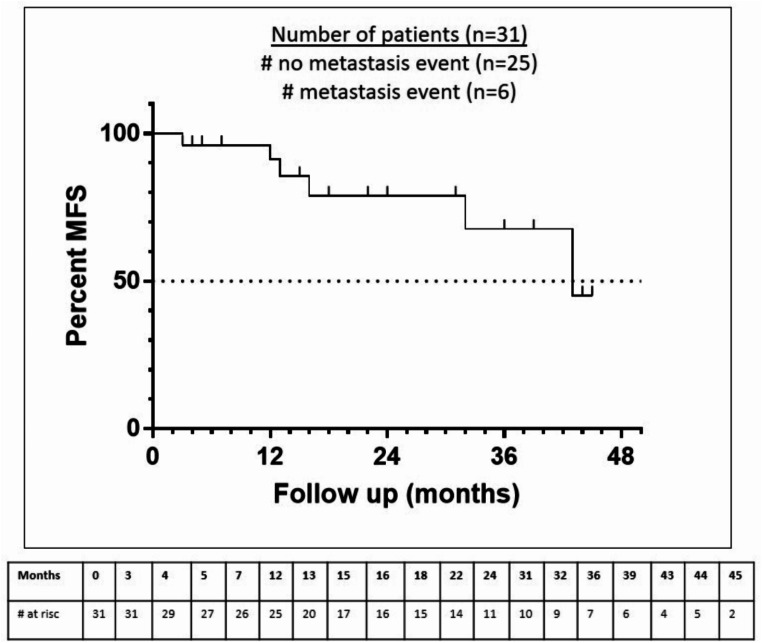
Median metastasis-free survival (MFS) was 43 months

PSA response of the cohort was observed at all time points. PSA ***of the cohort*** decreased about 91.3%, 95% and 98.1% after 3 months, 6 months and 9 months, resp. (see Fig. 1). ***Additional individual PSA reduction of ≥ 90% and ≥ 50% was achieved after 3 months in 74.2% and 90.3% of the patients resp.*** Initial PSA progression was seen in only 3.2%. PSA level decreased ≤ 0.2 ng/ml in 54.8% of patients ***after 3 month*** (see Fig. 2). During treatment 19.4% of patients showed progression to metastatic disease resulting in a median MFS of 43 months (see Fig. 3).

### Safety and subsequent therapy

In 32.3% of the patients no adverse events were documented. In 67.7% of the patients any adverse event, but only in 6.5% of them an adverse event grade ≥ 3 occurred. No death occurred. Table 2 presents a summary as well as most frequent types of adverse events. In 38.7% of the patients apalutamide dose reduction was necessary due to toxicity. 32.3% of the patients discontinued apalutamide treatment due to any reason: progression to metastatic disease was observed in 19.4%, toxicity in 6.5% and patients request in 9.7%. After discontinuation of apalutamide treatment due to progression 12.9% received abiraterone and 3.2% radiation therapy only. In 6.5% therapy was switched to enzalutamide due to toxicity.

**Table 2:** Summary of adverse events (AE) in general and the most frequent AE´s

	Patients with AE	Rash	Fatigue	Hot flash	Fall	Dizziness
*all grades [%]*	67.7	16.1	12.9	9.7	6.5	6.5
*grade ≥ 3 [%]*	6.5	3.2	3.2	0	0	0

## Discussion

Non-metastatic castration-resistant prostate cancer (nmCRPC) frequently advances to metastatic disease associated with poor clinical outcome as well as reduction of quality of life (Smith 2005 and 2011, Rönningas 2022). Recently the phase III trial Spartan demonstrated that apalutamide a novel receptor axis-targeted agent significantly improved metastasis-free survival (MFS) as well as overall survival (OS) in nmCRPC patients compared to placebo (Smith 2018 and 2021). However, due to lack of evidence it remains unclear if apalutamide in real-world clinical practice also results in improved outcome.

In our multicenter real-world study there are some important demographic differences: compared to Spartan trial our nmCRPC patients showed a higher ECOG PS (> 70% ≥ ECOG PS 1) and nearly 30% of the cohort a ECOG PS ≥ 2. Especially this patient group is even not included in the Spartan trial (Smith 2018). In addition real-world patients showed a higher proportion of high-grade Gleason score (57.9% vs. 43.5%) as well as PSADT ≤ 6 months (81.5% vs. 71.5%). Similar differences were described by Sánchez JC et al. (2023) in a smaller real-world nmCRPC cohort (*n* = 18). ***In Spartan trial number of pre-treated patients were nearly doubled compared to our cohort***,*** maybe explained by the larger number of patients.*** In both real-world and Spartan trial conventional imaging was performed in about 55% of the patients (Smith 2018). Nowadays there is a broader availability of PSMA-PET/CT as preferred and guideline-recommended staging imaging tool in nmCRPC reaching almost 100% detection rate of metastases. So using PSMA-PET/CT routinely therapy modalities of nmCRPC will change in the near future (Fendler 2019, Baboudjian 2022).

Our real-world cohort showed a rapid and durable PSA response. Median PSA levels ***of the cohort*** are decreasing with the lowest level after 9 months (0.1ng/ml). ***After 3 months a PSA reduction ≥ 90% and ≥ 50% was achieved in 74.2% and 90.3% resp.*** Deep PSA response reflected in a PSA-level ≤ 0.2ng/ml was obtained in 54.8% in real-world setting. Our data are in line with Spartan trial showing a PSA decrease ≥ 90% in 62% but a lower level of patients reaching PSA ≤ 0.2ng/ml (34%) despite higher percentage of PSADT ≤ 6months and high-grade Gleason score in our cohort. Nevertheless MFS was 43 months in our real-world cohort and ***comparable*** to the pivotal trial (40.5 months) (Smith 2018, Saad 2022).

Apalutamide treatment was also safe in real-world use. The documented overall AE rate (67.7%) as well as AE ≥ 3 (6.5%) was distinct lower compared to Spartan trial (96.5%, 45.1% resp.). In clinical trials reporting of AE´s are more strict and detailed compared to real-world practice (Eichler 2021). Additional patients maybe consult directly the specialist of the affected organ e.g. dermatologist in case of rash (Katsuta 2024). So the AE differences are not surprising and are in line with other real-world data (Sánchez JC 2023, Hussain 2022). Nevertheless beside the known and described side effects of apalutamide like rash and fatigue about 10% of the real-world patients mentioned hot flashes. In Spartan trial incidence of hot flashes are not reported as frequent side effect (> 15%) or adverse events of interest. Only a small number of patient (0.5%) in Spartan trial discontinued apalutamide therapy due to hot flashes (Smith 2018). Our data clearly identify the higher incidence and severity of hot flashes in a real-world population and should be considered when treating nmCRPC patients with apalutamide. Discontinuation of apalutamide treatment in real-world was also similar to Spartan trial (32.2% vs. 39.1%) as well as nmCRPC patients with disease progression (19.4% vs. 19.3%). Apalutamide dosage was reduced in 38.7% due to toxicity – reflecting the higher ECOG PS with more comorbidities however reaching satisfactory treatment efficacy.

Subsequent therapy in case of progression was abiraterone acetate (plus prednison) similar to the Spartan population. Another real-world study focusing ADT/apalutamide in nmCRPC patients (*n* = 18) used predominantly taxan-based chemotherapy (Sánchez JC 2023). This might depend on local reimbursement modalities and therapy restrictions. If AE´s were the reason for discontinuation a second hormonal agent was preferred because changing mode of action was not meaningful and other potential agents could be reserved for future use.

However our results are in line with the pivotal trial but there are several limitations of our real-world study. The small number of patients reflecting the increasing use of PSMA-PET/CT in the event of rising PSA-levels as well as the short follow-up. In Spartan trial the number of pre-treated patients were nearly doubled compared to our real-world cohort hamper the comparability of the findings. Another limitation is the missing standard of imaging to assess metastatic disease under treatment illustrating the typical limitations of real-world studies beside for instance medical records with unavoidable lack of data especially in a multicenter setting.

In conclusion in this largest nmCRPC real-world cohort more comorbid patients with a higher ECOG PS – not included in clinical trials- and more aggressive tumors were treated with apalutamide. Nevertheless efficacy results as well as adverse events are consistent with data of the phase III trial Spartan showing a rapid, durable and deep PSA response with similar MFS. ***As a matter of fact*** in real-world hot flashes are a ***clinical*** relevant and ***notable*** side effect.

## Data Availability

The data cannot be shared openly. Data of the findings have been deposited on the server of Urological Center Mittelhessen and can be aprehended after contacting the corresponding author.
